# Supplementary material for: Universal Virucidal Activity of Calcium Bicarbonate Mesoscopic Crystals That Provides an Effective and Biosafe Disinfectant
Source: Microorganisms. 2022 Jan 24;10(2):262. doi: 10.3390/microorganisms10020262 (PMC8877192; doi:10.3390/microorganisms10020262)
Supplement: Supplementary file 1 [file microorganisms-10-00262-s001.zip › microorganisms-1530970-supplementary.pdf]

Table S1. Primers used in this study

Virus	Target	Primer	Sequence (5' - 3')	Annealing temperature	Amplicon size (bp)	Reference
IBRV	gB	BoHV-F	TGTGGACCTAAACCTCACGGT	60°C	97	1
		BoHV-R	GTAGTCGAGCAGACCCGTGTC			
PrV	gB	PRV-gB718F	ACAAGTTCAAGGCCCACATCTAC	62 °C	94	2
		PRV-gB812R	GTCYGTGAAGCGGTTCGTGAT			
CHV-1	gB	CHV-For	ACAGAGTTGATTGATAGAAGAGGTATG	60°C	136	3
		CHV-Rev	CTGGTGTATTAAACTTTGAAGGCTTTA			
EHV-1	gB	FC3	ATACGATCACATCCAATCCC	60°C	188	4
		R1	GCGTTATAGCTATCACGTCC			
BAdV-7	DNA	BAdV-7-F	GCGTTTTGGACAACACTTCA	52 °C	200	This study
	polymerase	BAdV-7-R	TGGTTCATTTGCAACCAAAA			
CPV-2	VP2	CPV-F	TGGAACTAGTGGCACACCAA	56 °C	200	5
		CPV-R	AAATGGTGGTAAGCCCAATG			
BPIV-3	MP	BPI3F	TGTCTTCCAATAGATAGAGGGATAAAATT	60°C	111	6
		BPI3R	GCAATGATAACAATGCCATGGA			
BRSV	NP	BRSV-F	GGTCAAACTAAATGACACTTTCAACAAG	60°C	137	1
		BRSV-R	AGCATACCACACAACTTATTGAGATG			
CDV	NP	CDV-N-For	ACCGGAAATCGATGGAAGC	56 °C	67	7
		CDV-N-Rev	GTCCCAGGTTGACTGAGCATCT			
NDV	NP	Pla-rt13	CAACAATAGGAGTGGAGTGTCTGA	55 °C	145	8
		Pla-rt14	CAGGGTATCGGTGATGTCTTCT			
VSV	L	VSNJV-F	TGATTCAATATAATTATTTTGGGAC	52 °C	266	9
		VSNJV-R	AGGCTCAGAGGCATGTTCAT			
SARS-CoV-2	N	nCOV_N_F2	AAATTTTGGGGACCAGGAAC	55 °C	158	10
		nCOV_N_R2	TGGCAGCTGTGTAGGTCAAC			
BCoV	N	BCoV-N-F	TTCTCAGCAACCATCAGGAG	60 °C	149	This study
		BCoV-N-R	ACCCCTTAGCTTCAGTAGCC			
SwIV	M1	IAV-M1-F	AGATGAGTCTTCTAACCGAGGTCG	60°C	99	11
		IAV-M1.2-R	TGCAAAGACACTTTCCAGTCTCTG			
EqIV	NP	EIV-NP-For	GAAGGGCGGCTGATTCAGA	58 °C	63	12
		EIV-NP-Rev	TTCGTCGAATGCCGAAAGTAC			
BVDV-I &	5'-UTR	324	ATGCCCWTAGTAGGACTAGCA	55 °C	288	13
BVDV-II		326	TCAACTCCATGTGCCATGTAC			
BRBV	P3	BRBV-Pol-F	CTACGGTGTTTTCCGTCCAA	56°C	195	This study
		BRBV-Pol-R	TTGCCAAGAACATTGTGAAGA			
FCV	RNA	FCV-RT-F	GATGTTAGGGGCATTCCAGA	60°C	155	This study
	polymerase	FCV-RT-R	AGTGCCGCATCAATTGACAT			
BRoV	VP6	BRoV-G6-	CGCTAACTACGTCGAAACAGC	60 °C	200	This study
		BR0V-G6-	AGTTCCAGTTCTCTATGTATTCCG			
BuORV	L2	Pyc-L2- F1	TCTATCCGAAGATCCGTG	58 °C	146	This study
		Pyc-L2 -R1	ACAGAAGAGTCGAGTAACAGC			

References

1. Thonur, L.; Maley, M.; Gilray, J.; Crook, T.; Laming, E.; Turnbull, D.; Nath, M.; Willoughby, K. One-step multiplex real time RT-PCR for the detection of bovine respiratory syncytial virus, bovine herpesvirus 1 and bovine parainfluenza virus 3. *BMC Vet. Res.* **2012**, *8*, 37. https://doi.org/10.1186/1746-6148-8-37.
2. Ma, W.; Lager, K.M.; Richt, J.A.; Stoffregen, W.C.; Zhou, F.; Yoon, K.J. Development of real-time polymerase chain reaction assays for rapid detection and differentiation of wild-type pseudorabies and gene-deleted vaccine viruses. *J. Vet. Diagn. Invest.* **2008**, *20*, 440-447. https://doi.org/10.1177/104063870802000405.
3. Decaro, N.; Amorisco, F.; Desario, C.; Lorusso, E.; Camero, M.; Bellacicco, A.L.; Sciarretta, R.; Lucente, M.S.; Martella, V.; Buonavoglia, C. Development and validation of a real-time PCR assay for specific and sensitive detection of canid herpesvirus 1. *J. Virol. Methods* **2010**, *169*, 176-180. https://doi.org/10.1016/j.jviromet.2010.07.021.
4. Kirisawa, R.; Endo, A.; Iwai, H.; Kawakami, Y. Detection and identification of equine herpesvirus-1 and -4 by polymerase chain reaction. *Vet. Microbiol.* **1993**, *36*, 57-67. https://doi.org/10.1016/0378-1135(93)90128-t.
5. Streck, A.F.; Rüster, D.; Truyen, U.; Homeier, T. An updated TaqMan real-time PCR for canine and feline parvoviruses. *J. Virol. Methods* **2013**, *193*, 6-8. https://doi.org/10.1016/j.jviromet.2013.04.025.
6. Horwood, P.F.; Mahony, T.J. Multiplex real-time RT-PCR detection of three viruses associated with the bovine respiratory disease complex. *J. Virol. Methods* **2011**, *171*, 360-363. https://doi.org/10.1016/j.jviromet.2010.11.020.
7. Coleman, J.W.; Wright, K.J.; Wallace, O.L.; Sharma, P.; Arendt, H.; Martinez, J.; DeStefano, J.; Zamb, T.P.; Zhang, X.; Parks, C.L. Development of a duplex real-time RT-qPCR assay to monitor genome replication, gene expression and gene insert stability during in vivo replication of a prototype live attenuated canine distemper virus vector encoding SIV gag. *J. Virol. Methods* **2015**, *213*, 26-37. https://doi.org/10.1016/j.jviromet.2014.11.015.
8. Qiu, X.; Yu, Y.; Yu, S.; Zhan, Y.; Wei, N.; Song, C.; Sun, Y.; Tan, L.; Ding, C. Development of strand-specific real-time RT-PCR to distinguish viral RNAs during Newcastle disease virus infection. *Scientific World J.* **2014**, *2014*, 934851. https://doi.org/10.1155/2014/934851.
9. Hole, K.; Velazquez-Salinas, L.; Clavijo A. Improvement and optimization of a multiplex real-time reverse transcription polymerase chain reaction assay for the detection and typing of Vesicular stomatitis virus. *J. Vet. Diagn. Invest.* **2010**, *22*, 428-433. https://doi.org/10.1177/104063871002200315.
10. Shirato, K.; Nao, N.; Katano, H.; Takayama, I.; Saito, S.; Kato, F.; Katoh, H.; Sakata, M.; Nakatsu, Y.; Mori, Y.; Kageyama, T.; Matsuyama, S.; Takeda M. Development of genetic diagnostic methods for detection for novel coronavirus 2019 (nCoV-2019) in Japan. *Jpn. J. Infect. Dis.* **2020**, *73*, 304-307. https://doi.org/10.7883/yoken.JJID.2020.061.
11. Hoffmann, B.; Harder, T.; Lange, E.; Kalthoff, D.; Reimann, I.; Grund, C.; Oehme, R.; Vahlenkamp, T.W.; Beer, M. New real-time reverse transcriptase polymerase chain reactions facilitate detection and differentiation of novel A/H1N1 influenza virus in porcine and human samples. *Berl. Munch. Tierarztl. Wochenschr.* **2010**, *123*, 286-292. https://doi.org/10.2376/0005-9366-123-286.
12. Lu, Z.; Dubovi, E.J.; Zylich, N.C.; Crawford, P.C.; Sells, S.; Go, Y.Y.; Loynachan, A.T.; Timoney, P.J.; Chambers, T.M.; Balasuriya, U.B. Diagnostic application of H3N8-specific equine influenza real-time reverse transcription polymerase chain reaction assays for the detection of Canine influenza virus in clinical specimens. *J. Vet. Diagn. Invest.* **2010**, *22*, 942-945. https://doi.org/10.1177/104063871002200614.
13. Vilcek, S.; Herring, A.J.; Herring, J.A.; Nettleton, P.F.; Lowings, J.P.; Paton, D.J. Pestiviruses isolated from pigs, cattle and sheep can be allocated into at least three genogroups using polymerase chain reaction and restriction endonuclease analysis. *Arch. Virol.* **1994**, *136*, 309-323. https://doi.org/10.1007/BF01321060.
